# Lymph node assessment in cervical cancer: current approaches

**DOI:** 10.3389/fonc.2024.1435532

**Published:** 2024-11-11

**Authors:** Adriane Dheur, Athanasios Kakkos, Denis Danthine, Katty Delbecque, Frédéric Goffin, Elodie Gonne, Pierre Lovinfosse, Clémence Pleyers, Alain Thille, Frédéric Kridelka, Christine Gennigens

**Affiliations:** ^1^ Department of Gynecology and Obstetrics, University Hospital of Liège, CHU Liège, Liège, Belgium; ^2^ Department of Radiology, University Hospital of Liège, CHU Liège, Liège, Belgium; ^3^ Department of Pathology, University Hospital of Liège, CHU Liège, Liège, Belgium; ^4^ Department of Medical Oncology, University Hospital of Liège, CHU Liège, Liège, Belgium; ^5^ Department of Nuclear Medicine and Oncological Imaging, University Hospital of Liège, CHU Liège, Liège, Belgium; ^6^ Department of Radiotherapy, University Hospital of Liège, CHU Liège, Liège, Belgium

**Keywords:** cervical cancer, lymph node assessment, sentinel lymph node, imaging modalities, surgical staging

## Abstract

Cervical cancer (CC) is the fourth most common neoplasia in women worldwide. Although early-stage CC is often curable, 40 to 50% of patients are diagnosed at a locally advanced stage. Metastatic disease accounts for the principal cause of death. Lymph node (LN) status is a major factor impacting treatment options and prognosis. Historically, CC was staged based only on clinical findings. However, in 2018, imaging modalities and/or pathological findings were included in the International Federation of Gynecology and Obstetrics (FIGO) staging classification. In the last decades, LN status assessment has evolved considerably. Full pelvic lymphadenectomy used to be the only way to determine LN status. Currently, several options exist: surgery with full lymphadenectomy, sentinel lymph node (SLN) biopsy or imaging modalities such as computed tomography (CT), magnetic resonance imaging (MRI) and positron emission tomography (PET). Regarding surgery, the SLN biopsy technique has become a standard procedure in cases of CC, with indocyanine green (ICG) being the preferred dye. Pelvic MRI is a valuable imaging technique modality for the evaluation of pelvic LNs. In locally advanced or in early-stage disease with suspicious LNs on CT scans or MRI, PET/CT is recommended for assessment of nodal and distant status. The best strategy for LN assessment remains a highly controversial topic in the literature. In this article, we aim to review and compare the advantages and limitations of each modality, i.e. imaging or surgical (lymphadenectomy or SLN biopsy) approaches.

## 1 Introduction

Cervical cancer (CC) ranks fourth among global women’s neoplasia, with over 85% of cases in developing countries. In 2022, there were 660,000 new cases and 350,000 deaths worldwide. Survival rates vary by stage, with 5-year overall survival (OS) at 92%, 65%, and 17% for early, locally advanced, and metastatic disease, respectively (1). In the Western world there is wider access to healthcare resources, including screening and vaccination programs, leading to different incidence and mortality rates. Based on the European Cancer Information System, it is estimated that CC accounts for 2.4% of all new cancer cases and for 2.4% of all female cancer deaths in the 27 European nations. Moreover, CC ranks as the 11th most common cancer among women in Europe (2). According to the International Agency for Research on Cancer, CC is the 13th most frequent cancer among women in Belgium, with an annual average of 639 and 236 new cases and deaths, respectively (3).

The International Federation of Gynecology and Obstetrics (FIGO) updated CC staging in 2018, incorporating imaging and pathology. Lymph node (LN) involvement determines stage IIIC, with sub-stages based on LN location and size of metastases. According to the TNM 8 classification of malignant tumors, macrometastases (MACs), micrometastases (MICs) and isolated tumor cells (ITCs) are reported as pN1, pN1(mi) and pN0, respectively (4). MACs and MICs are thus included in the FIGO 2018 classification, whereas ITCs are not.

LN status influences treatment decisions, such as extended-field radiotherapy (RT), and the potential use of immunotherapy in the future.

Traditionally, lymphadenectomy was the only way to accurately determine the LN status. Currently, several options exist: LN dissection, imaging techniques, or both. Nevertheless, the best strategy to detect metastatic LNs is a very controversial topic, regarding the choice of a surgical or radiological approach.

## 2 LN assessment in early-stage CC

Early-stage CC refers to FIGO 2018 IA, IB1 and IB2 disease and is determined by the depth of stromal invasion and the maximum tumor diameter.

### 2.1 Risk of pelvic and para-aortic nodal involvement

As tumor invasion depth increases, pelvic LN (PLN) metastasis likelihood rises, from 2.1% in stage IA1 to 3.9% in IA2 (2). Early-stage CC has typically < 5% para-aortic (PAo) LN metastases (5).

Lymphovascular space invasion (LVSI) strongly predicts positive LN status. In stage IB (FIGO 2009), LVSI presence correlates with LN involvement (9.3% vs. 1.7% without LVSI) (6). Stage IA1 rarely involves LN metastases, unless LVSI is present. Thus, according to the European Society of Gynaecological Oncology (ESGO)/the European Society for Radiotherapy and Oncology (ESTRO)/the European Society of Pathology (ESP) guidelines, LN staging is not indicated in T1a1 LVSI-negative patients, but can be considered in T1a1 LVSI-positive patients (7). This is true for both squamous cell and adenocarcinoma histology (8).

Tumor size is also predictive; ≤2 cm, 2–4 cm, and ≥4 cm tumors have LN involvement rates of 6%, 18.4%, and 36.4%, respectively.

Du et al.’s study assessed LN metastasis in stage IB (FIGO 1999) patients undergoing radical hysterectomy associated with a pelvic and PAo lymphadenectomy. Radiological methods [computed tomography (CT), magnetic resonance imaging (MRI), positron emission tomography (PET)/CT (PET/CT)] identified PLN, common iliac, and PAo LN metastases in 13.5%, 3.4%, and 4.7% of cases, respectively (9).

Wenzel et al. aimed to develop a risk stratification tool to identify women with early-stage CC (FIGO 2009 stages IA2, IB1, and IIA1) at low risk of LN metastasis. The authors analyzed clinicopathological risk factors in a substantial cohort of women from Denmark, Sweden, and the Netherlands. The authors found that LVSI was the most significant risk factor for LN metastasis, followed by tumor size of 21–40 mm and depth of invasion greater than 10 mm (10). This risk stratification tool provides key information, which assists in the shared decision-making process regarding LN dissection.

### 2.2 Pelvic region assessment

The pelvic lymphatic region includes the obturator, the external and internal iliac, the lower portion of the common iliac, the sacral and the para-rectal nodes.

The obturator and external iliac LNs are the most frequently invaded regions, while about 10% of the positive nodes are located in the presacral or internal iliac areas, and sometimes even above the aortic bifurcation.

#### 2.2.1 Imaging

Imaging techniques to evaluate the nodal status include CT, MRI, PET, PET/CT, PET/MRI. The use of ultrasonography (US) will not be discussed here as it is of limited use for evaluating LN status, nor lymphangiography, which is only of historical interest.

##### 2.2.1.1 Computed tomography

In the literature the accuracy of CT scans in detecting metastatic LNs varies from 37% to 86% (11). CT uses size criteria to evaluate nodal status: a LN is considered pathological (malignant) and measurable when its smallest axis is ≥ 10 mm (the smallest axis being the axis perpendicular to the largest axis of the LN) (12).

However, normal sized nodes can contain microscopic metastases, leading to false-negative (FN) results, while enlarged nodes due to inflammation or hyperplastic changes can give false-positive (FP) information.

In conclusion, CT has a limited role in accurately detecting metastatic LNs (11).

##### 2.2.1.2 Magnetic resonance imaging

MRI is crucial for assessing tumor characteristics and LN status in CC, including extension into nearby structures and LN involvement. Pelvic MRI complements clinical examination, except in the case of T1A tumors with free margins post-conization (7).

While MRI shows high specificity (up to 96.8%) for nodal metastases, its sensitivity for PLNs is only 50%, compared to 66.6% for PAo LNs (13, 14). Functional MRI techniques such as dynamic contrast-enhanced (DCE) or diffusion-weighted imaging (DWI) improve nodal assessment, particularly by detecting larger metastatic LNs with decreased apparent diffusion coefficient (ADC) (15, 16).

However, both metastatic and non-pathological nodes may show diffusion restriction. Additional T1 and T2 weighted imaging aids LN morphology evaluation, with criteria for suspicious enlargement varying by location (17).

A meta-analysis reported DWI sensitivity and specificity at 0.86 and 0.84 respectively, with significant study heterogeneity (18).

In 2023, Shakur et al. highlighted MRI’s critical role in CC management, aiding treatment decisions by accurately assessing tumor size, parametrial involvement, and LN status (17).

##### 2.2.1.3 Positron emission tomography/computed tomography with 2-deoxy-2-[fluorine-18]fluoro-D-glucose

Driscoll et al. showed PET/CT to be of limited value in their cohort of patients with early stage (stages IA–IB1) MRI LN negative CC (19). Signorelli et al. evaluated 159 women with stages IB1 and IIA CC, and demonstrated that PET/CT had low sensitivity (32.1%) for LN involvement and did not change treatment planning (20). This low sensitivity is consistent with a more recent and larger series demonstrating a sensitivity of 10–35% (21).

In conclusion, the clinical and cost benefits of PET/CT are limited when CT or MRI is negative.

#### 2.2.2 Surgery

Historically, it was thought that CC’s LN spread was almost invariably from the pelvic wall to the common iliac and then PAo nodes. More recent studies, including those using the sentinel lymph node (SLN) mapping technique, highlight that any of the PLNs, and even the common iliac and low PAo LNs, may be the first site of LN metastasis.

Classically, PLN dissection includes bilateral removal of nodal tissue from the distal one-half of each common iliac artery, the anterior and medial aspect of the proximal half of the external iliac artery and vein, and the distal half of the anterior obturator fat pad to the obturator nerve (22, 23).

##### 2.2.2.1 Full pelvic LN dissection (PLND) or SLN biopsy?

###### 2.2.2.1.1 Definitions and rationale

While PLND can give complete pathological nodal sample evaluation, it carries the risk of both intra- and postoperative complications. Intraoperative complications include damage to neurovascular and ureteral structures, increased blood loss (and thus blood transfusions), but also increased surgical time. Postoperative risks include infection, venous thromboembolism, and lymphoedema or lymphocysts (24).

More recently, the SLN biopsy technique has been widely studied and has become the standard of care for CC. Following the 2023 ESGO/ESTRO/ESP guidelines, SLN biopsy before pelvic lymphadenectomy should be performed (7). Its advantages include easy cervix accessibility, straightforward dye injection, reduced operative time and morbidity compared to routine pelvic lymphadenectomy, thereby lowering leg lymphoedema risk. Furthermore, by detecting LNs in uncommon locations and identifying MICs and ITCs by ultrastaging, the SLN technique can enhance the detection rate of LN metastases (23). Indeed, Smits et al. demonstrated that in a population of 100 women with early-stage CC undergoing the SLN procedure, 37% of SLNs were found in unusual locations (27% in common and unusual locations, 10% in unusual locations only). Factors linked to higher incidences of unconventional nodal locations include lower body mass index, nulliparity and tumor size above 20 mm (25).

In a recent meta-analysis investigating the impact of SLN biopsy alone versus PLND on survival in patients with early-stage CC, both 5-year disease-free survival (DFS) and OS rate after SLN biopsy alone were greater than 90% and did not vary from PLND survival data. These results suggest that SLN biopsy could be considered the standard surgical procedure to reduce postoperative complications in patients with early-stage CC in order to improve quality of life (QoL) and prognosis, but further studies are needed to confirm these findings (26, 27).

###### 2.2.2.1.2 SLN technique

The combination of technetium-99 (99mTc) and blue dye has been widely used, but recently there has been increasing interest in the use of fluorescent dyes such as indocyanine green (ICG), which could improve the SLN detection rate. According to the 2023 ESGO/ESTRO/ESP guidelines, ICG is the most efficient technique. A combination of blue dye with radiocolloid remains an alternative technique (7).

A 2016 meta-analysis by Ruscito et al. compared ICG to blue dye, with or without radioisotopes, demonstrating that ICG significantly improved the overall and bilateral SLN detection rates compared to blue dye; ICG was equivalent to radioisotopes alone or combined with blue dye (28). Moreover, the prospective randomized FILM trial found superior SLN detection rates with ICG compared to blue dye in patients with endometrial and cervical cancers (97% vs. 47%) (29).

Furthermore, Lührs et al. showed that combining ICG and 99mTc did not improve the bilateral detection rate of SLNs in CC (30). Of note, the COMBITEC study conclusions were identical, but in early-stage endometrial cancer (31).

In conclusion, according to the 2023 ESGO/ESTRO/ESP guidelines, ICG is the preferred technique, but a combination of blue dye with technetium remains an alternative approach (7).

###### 2.2.2.1.3 Accuracy

The diagnostic accuracy of SLN mapping is crucial in reducing FN rates, as nodal status is a major factor impacting treatment options and prognosis. The goal is thus bilateral SLN detection in the pelvic region (32).

The Memorial Sloan Kettering Cancer Center (MSKCC) team created an algorithm for SLN mapping, known as the “MSKCC criteria”, incorporating SLN mapping into the surgical treatment of early stage CC, ensuring that LN metastases are accurately detected but minimizing the need for complete lymphadenectomy. Steps include: SLN removal and, in case of negativity on routine hematoxylin and eosin (H&E) staining, ultrastaging; removal of any suspicious LNs; side-specific LN dissection including inter-iliac or subaortic nodes if there is no mapping on a hemi-pelvis, and parametrectomy en bloc with resection of the cervical tumor (33, 34).

This approach significantly decreases FN incidence compared to the removal of colored nodes only (33). Tax et al.’s meta-analysis shows FIGO IA2, IB1, IIA primary tumor size <40 mm patients with no suspicious LNs, bilateral negative SLN post-ultrastaging, have a residual 0.08% (1/1257) occult metastasis risk under MSKCC criteria. Therefore, this study does not recommend full PLND in these cases (35).

SENTICOL I, a multicenter prospective longitudinal study from January 1, 2005, to June 30, 2007, assessed the sensitivity of SLN biopsy and negative predictive value (NPV) in 145 women with FIGO 1999 stage IA1 with LVSI to stage IB1. Using 99mTc lymphoscintigraphy and blue dye injection, surgeons proficient in SLN biopsy conducted laparoscopic LN mapping, SLN removal, and LN dissection. Routine staining was performed on both SLNs and non-SLNs, with negative SLNs undergoing ultrastaging. The study found combined labeling SLN detection to be effective, with over 95% SLN detection rate and bilateral detection in 76% of patients. It also demonstrated high sensitivity (92%) and NPV (98.2%), emphasizing the importance of bilateral detection for reliability (32).

###### 2.2.2.1.4 Morbidity

The SENTICOL II trial focused on the morbidity associated with PLND. Patients with FIGO 2009 stage IA2 to IIA1 were randomized between SLN biopsy alone and SLN plus PLND, both followed by radical surgery (radical hysterectomy or trachelectomy). The primary endpoint was morbidity related to the LN dissection, while 3-year recurrence-free survival (RFS) was a secondary endpoint. The results revealed that SLN biopsy alone was associated with a lower rate of early complications, and improved QoL. The RFS was similar in both arms of the study (36).

###### 2.2.2.1.5 Ongoing trials

Two prospective trials evaluating long-term oncological outcomes after SLN biopsy in early stage CC are ongoing and their results, if positive, would support the definitive implementation of the SLN approach.

SENTICOL III is an international, randomized, multicenter, single-blind trial. It compares SLN biopsy alone versus SLN biopsy plus PLND in terms of DFS and health-related QoL in patients with negative SLNs (27).

The PHENIX trial is another multicenter randomized trial that aims to evaluate the oncological outcomes of SLN biopsy alone versus PLND. The primary endpoint is DFS (37).

###### 2.2.2.1.6 Frozen section examination

The FSE technique is controversial due to its poor sensitivity in detecting small metastases as ITCs and MICs. Indeed, sensitivity ranges from 42.3% to 87.5%, and increases from 56.4% to 88.9% when excluding ITCs (38–43). According to the 2023 ESGO/ESTRO/ESP guidelines, SLNs from both sides of the pelvis and any suspicious LNs should be assessed intra-operatively by frozen section (level of evidence III, recommendation grade A). Post-FSE, all SLNs should be processed according to the pathological protocol for ultrastaging (7). The “pathological” challenge is a balance between FSE accuracy, requiring substantial sampling, and the need to keep a sufficient number of slides for effective ultrastaging techniques (44).

###### 2.2.2.1.7 Ultrastaging

SLN biopsy allows nodal ultrastaging. This technique combines serial sectioning and immunohistochemistry (IHC) and increases the detection of low-volume disease, such as MICs or ITCs that would not have otherwise been identified with routine histological examination and conventional staining.

The SLN must undergo complete processing, with precise cutting into 2-mm sections perpendicular to its length. If the initial assessment with H&E staining reveals no abnormalities, the ultrastaging procedure entails four levels spaced at 150-µm intervals. Four slides are obtained from each level: one with H&E staining, one with IHC staining, and two slides remaining unstained. Additionally, IHC is performed using the cytokeratin AE1/3 antibody (45).

Multiple different ultrastaging protocols are in endometrial and cervical cancers (46). Two main macroscopic slicing methods exist. The bread-loaf technique involves slicing the LN transversely into multiple thin sections, perpendicular to the long axis; LNs are cut into slices of uniform thickness, usually 2-3 mm, and each slice is examined histologically (47). It ensures that the entire node is sampled, increasing the chances of detecting small metastases, and provides a comprehensive view of the LN architecture, which is beneficial for the identification of small clusters of cancer cells. On the other hand, the longitudinal sectioning method involves slicing the LN longitudinally, along its longest axis. It helps to conserve the nodal architecture and may be useful in detecting metastases that are aligned along the node’s long axis (46). According to the latest ESGO/ESTRO/ESP guidelines, bread-loaf slicing is preferred for ultrastaging LN in CC; indeed, the ultrastaging procedure should include a minimum of five serial sections at 200 µm (7).

SENTIX is an international, multicenter, prospective cohort study evaluating SLN biopsy without PLN dissection in patients with early stage CC. The primary endpoint is the recurrence rate at 24 months after surgery. The null hypothesis is that the recurrence rate after SLN biopsy is non-inferior to the reference recurrence rate (7% in patients after systematic PLND), but that the SLN technique is associated with significantly lower postoperative morbidity. This study showed that the bilateral SLN detection rate reached 92.3% with the median of 3 SLNs per patient. The majority (97.3%) were localized in the pelvic level I, below the interiliac bifurcation. There was a low rate (1.3%) of isolated positive SLNs in pelvic level II (the common iliac and the presacral regions) and no SLNs in the low PAo region. No laterally distinct distribution of SLNs was found. This trial also revealed that, in early stage CC patients, SLN biopsy with pathological ultrastaging and no further lymphadenectomy is not associated with an increased risk of recurrence (48).

This strategy increases the prevalence of patients with positive LNs by up to 15% and improves LN staging, especially when SLNs are detected in both hemipelvises (49).

However, it is important to keep in mind that the use of ultrastaging does not significantly impact 5-year DFS, as demonstrated by Parpinel et al. in 2023 (26).

##### 2.2.2.2 Radical surgery and LN staging: one or two successive operative steps?

Three surgical algorithms for SLN staging exist: one-step without intraoperative SLN assessment, one-step with FSE of SLNs, and a two-step strategy with definitive surgery after SLN ultrastaging results (38, 41, 50). Of note, knowing LN status during surgery is crucial for immediate treatment decisions, to prevent morbidity from combining radical surgery and adjuvant pelvic RT (51, 52).

The 2023 ESGO/ESTRO/ESP guidelines favor the one-step procedure with FSE, ideally using ICG dye for bilateral SLN detection, followed by complementary ultrastaging analysis. However, due to the poor accuracy of FSE in detecting low-volume metastases, a precise and rigorous pathological protocol is essential (42, 51, 53).

### 2.3 Para-aortic region assessment

The incidence of PAo LN involvement increases with tumor “T” stage. However, due to a low risk of PAo LN involvement in early stage CC, routine PAo LN dissection (PALND) is not recommended (54–56).

If any LN involvement is detected intraoperatively, PALND at least up to the inferior mesenteric artery (IMA) may be considered for staging purposes, before referring patients for definitive concurrent chemoradiotherapy (CCRT) (7).

## 3 LN assessment in locally advanced CC (LACC)

LACC refers to FIGO 2018 stage T1B3 (tumor confined to the cervix with a tumor > 4 cm in greatest dimension) to T4A (tumor invading the mucosa of the bladder or rectum, or extends beyond the true pelvis to adjacent pelvic organs).

In the LACC setting, PLNs are involved in about 30–50% of cases, and PAo LNs may be involved in 10–35%. The rate of clinically occult PAo LN metastases rises from 12% to 22% in the presence of affected PLNs (57).

Staging of LACC patients can be achieved using imaging techniques such as ^18^F-FDG-PET/CT; however, regarding PAo LN assessment, FN rates can be as high as 20%, particularly in patients with positive PLN metastases. In this context, surgical staging allows patients with microscopic PAo LN metastases to be identified. This is of major importance for LACC treatment based on chemoradiation followed by brachytherapy (7). Indeed, PAo LN involvement leads to an extended-field RT, and in the near future to the addition of pembrolizumab, as in the ENGOT-cx11/KEYNOTE A18 trial (57–59).

The role of neoadjuvant chemotherapy (NACT) in CC is still under study, and it is not universally accepted as standard care.

A study by Mereu et. al., as well as the EORTC 55994 trial, compared NACT followed by surgery with concurrent chemoradiation; it provided mixed results and highlighted the need for patient-specific treatment decisions (60, 61).

On the other hand, findings from the GCIG INTERLACE trial revealed that patients with LACC who received induction chemotherapy before CCRT experienced higher PFS and OS rates at 5 years, compared to those who underwent CCRT alone​ (62).

### 3.1 Risk of pelvic and PAo LN involvement

The risk of PAo LN involvement increases as the local disease extent increases; pathologically confirmed PAo disease after surgical staging is present in 21% of cases in stage IB3 and rises up to 35% in stage IIIB (63, 64).

In a 2017 prospective randomized trial, 234 patients with FIGO 2009 stage IIB-IVA CC were randomly assigned to surgical staging (arm A) or to clinical staging and primary chemoradiation (arm B). Arm B patients underwent CT-guided biopsy of suspicious PAo LNs. Confirmed PAo metastasis patients received extended-field RT. Pelvic and PAo LN metastases were identified after surgical staging in 51% and 24% of patients, respectively (65). There was no data on survival outcomes described in this study.

### 3.2 Pelvic region assessment

PLN status is evaluated by MRI and also by ^18^F-FDG-PET/CT. This status is very useful in deciding the best strategy regarding PAo LN assessment. It also supports the practice of external beam radiation boost directed towards positive PLNs at the initial imaging work-up (64, 66, 67).

### 3.3 PAo region assessment

PAo LN detection is a crucial point as it can result in upstaging, thus in modifying the treatment plan with extended-field RT. Nevertheless, the best strategy for detection of PAo LNs remains controversial, regarding whether to perform a surgical versus a radiological staging.

#### 3.3.1 Imaging

##### 3.3.1.1 ^18^F-FDG-PET/CT

Whole body ^18^F-FDG-PET/CT is clearly the preferred imaging modality for detecting LN metastases in LACC patients (68, 69); several meta-analyses have shown its diagnostic performance superiority over CT and MRI (70, 71).

PLN uptake is the most significant factor for predicting PAo LN extension (72), with this risk increasing with the number of PLNs involved (5, 66, 67).

Although highly specific for detecting metastatic PAo LNs, ^18^F-FDG-PET/CT has an FP rate of 6% (73), related to FDG uptake by inflammatory nodes or misinterpretation of physiological FDG uptake in the ovary, intestine or urinary tract (74); a FN rate of 12% (57, 70, 75), which doubles to 22% for patients with PET/CT positive PLNs due to its limited spatial resolution (about 5 mm) is described (73).

More recently, volume-based metabolic parameters such as metabolic tumor volume (MTV) and total lesion glycolysis (TLG) have been adopted to measure the metabolic activity of the primary tumor, or the entire LN (67, 76, 77). Each 10 cm³ increase in MTV has been associated with a 1.24 higher risk of PAo LN involvement (66).

The 2023 ESGO/ESTRO/ESP guidelines recommend PET/CT for assessing nodal and distant disease in cases of suspicious LNs on imaging (types of imaging technique not specified) (7).

Despite its FN rate in cases of positive PLNs, ^18^F-FDG-PET/CT remains the most accurate imaging tool for evaluating PAo extension in LACC (66).

##### 3.3.1.2 PET-MRI

PET/MRI is a new modality that combines the advantages of local staging of MRI LN detection by PET. PET/MRI could significantly improve LN detection compared to PET/CT. In a study published in 2021 by Zhu et al., the sensitivity, specificity, and accuracy of PET/MRI in the diagnosis of LNs were 94.74%, 93.33% and 93.88%, respectively. These results show that PET/MRI is more accurate than the other imaging modalities (78). However, its use is not currently a common practice.

#### 3.3.2 Surgery

##### 3.3.2.1 Purpose

The risk of PAo LN involvement is correlated with the metabolic PLN metabolic status.

A phase II single-arm trial on 237 patients with ^18^F-FDG-PET/CT negative PAo LNs, who benefited from laparoscopic staging, demonstrated a 12% false discovery rate for the detection of positive PAo LNs using metabolic imaging. Moreover, Gouy et al. reported identical survival rates in patients with PAo LN metastases of < 5 mm detected surgically and in patients without PAo LN involvement, suggesting a potential impact of lymphadenectomy on survival (72, 79).

In a study including 125 patients with negative PAo LN uptake on ^18^F-FDG-PET/CT who underwent PALND, Martinez et al. showed a FN rate of 27.7% and 5.1% in patients with and without ^18^F-FDG-PET/CT pelvic LN uptake, respectively. In fact, the risk of PAo involvement increases with the number of suspicious PLNs and with the increasing values of PLN metabolic parameters. The author concluded that the risk of PAo LN metastasis in cases of patients with ^18^F-FDG-PET/CT negative pelvic status is very low, so PALND does not appear to be justified. In contrast, for patients with preoperative PLN uptake on ^18^F-FDG-PET/CT, surgical staging led to treatment modification in more than 25% of cases and should therefore be performed (66). De Cuypere et al. confirmed these data in a retrospective study (67).

According to the 2023 ESGO/ESTRO/ESP guidelines, PALND (at least up to the IMA) may be used to evaluate the need for elective PAo external beam RT in patients with negative PAo LNs and positive PLNs on imaging (level of evidence IV, grade of recommendation C). If PALND is not performed, the indication for elective PAo irradiation can be based on the number of level I positive nodes on imaging (external iliac, interiliac, internal iliac, e.g. >2 positive nodes). Moreover, elective PAo radiation should always be applied in patients who have even one positive node at level II (common iliac), and above, on imaging (7).

Surgical removal of large pathological pelvic and/or PAo LNs before definitive CCRT is not routinely recommended (7).

Of note, only 2 randomized controlled trials have been performed to address the question of radiological-clinical or surgical PAo LN evaluation. The Lai et al. study was prematurely closed and limited by numerous methodological biases. The UTERUS-11 is a study that compared surgical and radiological LN staging in 225 FIGO 2009 IIB-IVA patients, with a median follow-up of 90 months. The author showed that OS and PFS were not statistically different, whereas cancer-specific survival (CSS) favored the surgical approach. Moreover, surgical staging, that included complete pelvic and PAo LN dissection, was safe and neither delayed CCRT nor increased complications (80).

The results of the Lymphadenectomy in Locally Advanced Cervical Cancer Study (LiLACS) are pending, but this randomized phase III trial has been definitively closed. Patients with ^18^F-FDG-PET/CT positive pelvic and negative PAo LNs were randomized into 2 groups: pelvic CCRT versus laparoscopic extraperitoneal PAo lymphadenectomy and tailored CCRT, with OS as the primary endpoint. Results from already accrued patients may clarify the role of prophylactic treatment of PAo LNs in patients at high risk of subclinical involvement (81).

##### 3.3.2.2 Morbidity

When retroperitoneal PALND is performed by experienced teams, both the risk of grades 3-4 intraoperative morbidity, and the proportion of patients whose CCRT is delayed by more than 30 days after surgery, are less than 5% (82). The most frequent early postoperative causes of morbidity are lymphatic complications such as lymphocele and lymphoedema. A study showed that lymphocysts occurred in 21.4% and 20.6% of cases when an inframesenteric and infrarenal lymphadenectomy (IM/IR-LND) was performed, respectively (83).

##### 3.3.2.3 Surgical approach for PALND

Debate persists regarding the upper extent of lymphadenectomy, with some favoring dissection to the left renal vein and others to the origin of the IMA (84). Petitnicolas et al.’s study found shorter operating times with inframesenteric lymphadenectomy and similar complication rates (83). Limited IMA dissection proponents argue that supramesenteric LN metastases, thus above the IMA, without involvement below are rare, with 1.36-3.3% of isolated supramesenteric metastases (84–86). Nevertheless, Gil-Moreno et al. reported that inframesenteric aortic nodes are negative in the presence of positive infrarenal nodes in about one third of patients with LACC and aortic metastases (87). Both ESGO/ESTRO/ESP and National Comprehensive Cancer Network (NCCN) guidelines recommend PALND at least up to the IMA for staging purposes (7, 88).

Historically, staging was done via transperitoneal laparotomy, but due to high morbidity (28-30%) and mortality (6-22%) rates it was replaced by transperitoneal laparoscopy in the 1990s (57); later, the laparoscopic extraperitoneal (retroperitoneal) approach was adopted (89, 90). The laparoscopic procedure has a complication rate of 4% to 18%, with lymphocysts being the most common issue (58). The transperitoneal method offers a larger working area and familiar landmarks but may require bowel mobilization. The extraperitoneal technique, while offering reduced bowel injury and adhesions and being feasible despite previous surgery, offers a limited working space and few landmarks, increasing the risk of disorientation (91). The 2023 ESGO/ESTRO/ESP guidelines do not endorse a preferred surgical method. Of note, SLNs are frequently found below the common iliac bifurcation, limiting this technique’s usefulness in detecting PAo LN metastases (48, 92).

## 4 Fertility sparing treatment

Recent studies highlight the critical role of a multidisciplinary approach in CC management, especially in the case of fertility-sparing treatment (FST). Fertility-sparing procedures include conization, simple trachelectomy, radical (vaginal) trachelectomy and abdominal radical trachelectomy. FST is a viable alternative to radical hysterectomy for young patients with squamous cell carcinoma or HPV-related adenocarcinoma <2 cm and negative PLNs, who wish to preserve their fertility.

Essential imaging tests, such as pelvic MRI and/or expert US, are necessary to assess the cervical length before (upper tumor-free margin) and after cone biopsy. Negative PLN status is essential for FST eligibility, making PLN staging the initial and crucial step in the process (excluding T1a1 LVSI negative patients). Identification and ultrastaging of SLNs is highly recommended. Any suspicious LNs, other than SLNs, found during surgery should be removed and sent for FSE. If SLNs cannot be detected on either side of the pelvis, a PLND should be performed on that side. If PLN involvement is detected during surgery, FST should be discontinued in favor of concurrent chemoradiotherapy and brachytherapy.

Additionally, the phase II CONTESSA/NEOCON-F trial aims to evaluate NACT followed by fertility-sparing surgery in FIGO 2018 stage IB2 CC patients who desire to preserve fertility (7, 93).

## 5 Best imaging during pregnancy

Ionizing imaging procedures, especially in early pregnancy, should be avoided due to potential risks on fetal development. CT scans are generally not recommended but could be used in cases of absolute necessity (7, 94). Non-ionizing methods such as US and MRI are preferred for evaluation of tumor characteristics and LN involvement, although gadolinium-enhanced MRI is not advised due to associated risks of stillbirth (94).

Whole-body diffusion-weighted MRI (WB-DWI/MRI) is a suitable alternative to ^18^F-FDG-PET/CT (that should be avoided) for detecting metastases without harming the fetus, and can also eliminate the need for gadolinium contrast and radiation for nodal and distant staging during pregnancy (7, 94).

Moreover, Ishiguro et al. support the use of MRI, especially without contrast, for managing CC in pregnant patients while ensuring fetal safety (95).

## 6 Artificial intelligence and radiomics

Artificial intelligence (AI) is a field of computer science and engineering focused on creating machines that can replicate human thinking and behavior to perform a series of tasks. AI technology primarily relies on machine learning (ML) and deep learning (DL) techniques. ML involves training models to optimize performance metrics using example data. DL, a newer sub-category of ML, is able to automatically learn representations of data, with neural networks being a key method (96, 97). The term “radiomics” refers to the automated identification of unique prognostic and diagnostic features in cancer imaging data. Radiomics focuses on enhancing image analysis through automated high-throughput extraction of numerous quantitative features from medical images (98).

A 2022 meta-analysis by Li et al. assessed the diagnostic performance of MRI-based radiomic features for preoperative prediction of LN metastasis in CC patients. The study reported good prediction performance, with an overall AUC of 0.83, sensitivity of 80%, and specificity of 76%. These findings indicate that MRI-based radiomic features can improve the accuracy of predicting LN metastasis. However, well-designed prospective studies with strict standardization of radiomics protocols are needed to validate its diagnostic performance and develop this technique for daily clinical use (99).

Recently, Lucia et al. focused on creating ML models to predict PAo LN involvement in patients with LACC prior to chemoradiotherapy by utilizing ^18^F-FDG-PET/CT and MRI radiomics. Their findings indicate that radiomic features significantly surpass clinical parameters in guiding the decision to undertake PAo LN staging or extended-field RT (100).

## 7 Conclusion

In early-stage CC, pelvic MRI is essential for the initial diagnostic work-up. In terms of surgical approach, SLN biopsy with ICG is the standard of care, with the SLNs subjected to FSE. SLNs need to be found per hemipelvis; otherwise a side-specific PLND is necessary. The MSKCC criteria ensure accurate LN metastasis detection while minimizing the need for complete lymphadenectomy.

In LACC, identifying PAo LNs is crucial for treatment planning, with ^18^F-FDG-PET/CT considered the most accurate imaging tool for evaluating PAo extension. Surgical staging involves PALND using a minimally invasive technique, with debates over the extent of dissection and preferred approach. In the LACC setting, assessing LN status involves a complementary approach, combining both imaging and surgery.

There are still several unresolved questions as to the impact of radiomic techniques.

## References

[1] Bray F Laversanne M Sung H Ferlay J Siegel RL Soerjomataram I. Global cancer statistics 2022: GLOBOCAN estimates of incidence and mortality worldwide for 36 cancers in 185 countries. CA: A Cancer J Clin. (2024) 74:229−63. doi: 10.3322/caac.21834 38572751

[2] European Cancer Information System. Available online at: https://ecis.jrc.ec.europa.eu/factsheets_2022.php (accessed August 01, 2024).

[3] Homepage – IARC. Available online at: https://www.iarc.who.int (accessed August 01, 2024).

[4] Union for International Cancer Control. TNM classification of Malignant tumours. 8th ed. Union for International Cancer Control (2017). Available online at: https://www.uicc.org/resources/tnm-classification-malignant-tumours-8th-edition (accessed July 23, 2024).

[5] Han X Wen H Ju X Chen X Ke G Zhou Y. Predictive factors of para-aortic lymph nodes metastasis in cervical cancer patients: a retrospective analysis based on 723 para-aortic lymphadenectomy cases. Oncotarget. (2017) 8:51840−7. doi: 10.18632/oncotarget.16025 28881693 PMC5584294

[6] Wenzel HHB Van Kol KGG Nijman HW Lemmens VEPP van der Aa MA Ebisch RMF. Cervical cancer with ≤5 mm depth of invasion and >7 mm horizontal spread — Is lymph node assessment only required in patients with LVSI? Gynecol Oncol. (2020) 158:282−6. doi: 10.1016/j.ygyno.2020.04.705 32381363

[7] Cibula D Raspollini MR Planchamp F Centeno C Chargari C Felix A. ESGO/ESTRO/ESP Guidelines for the management of patients with cervical cancer – Update 2023*. Int J Gynecol Cancer. (2023) 33:649−66. doi: 10.1136/ijgc-2023-004429 37127326 PMC10176411

[8] Reynolds EA Tierney K Keeney GL Felix JC Weaver AL Roman LD. Analysis of outcomes of microinvasive adenocarcinoma of the uterine cervix by treatment type. Obstet Gynecol. (2010) 116:1150−7. doi: 10.1097/AOG.0b013e3181f74062 20966701

[9] Du R Li L Ma S Tan X Zhong S Wu M. Lymph nodes metastasis in cervical cancer: Incidences, risk factors, consequences and imaging evaluations. Asia Pac J Clin Oncol. (2018) 14:e380−5. doi: 10.1111/ajco.2018.14.issue-5 29855154

[10] Wenzel HHB Hardie AN Moncada-Torres A Høgdall CK Bekkers RLM Falconer H. A federated approach to identify women with early-stage cervical cancer at low risk of lymph node metastases. Eur J Cancer. (2023) 185:61−8. doi: 10.1016/j.ejca.2023.02.021 36965329

[11] Meva J Chaudhary RK Bhaduri D Bhatia M Hatti S Ba R. Lacunae in International Federation of Gynecology and Obstetrics (FIGO) classification for cervical carcinoma: observational study using TNM classification as comparator. Int J Gynecol Cancer. (2013) 23:1071−7. doi: 10.1097/IGC.0b013e31829783c4 23792602

[12] Fischerova D Frühauf F Burgetova A Haldorsen IS Gatti E Cibula D. The role of imaging in cervical cancer staging: ESGO/ESTRO/ESP guidelines (Update 2023). Cancers (Basel). (2024) 16:775. doi: 10.3390/cancers16040775 38398166 PMC10886638

[13] Bourgioti C Chatoupis K Rodolakis A Antoniou A Tzavara C Koutoulidis V. Incremental prognostic value of MRI in the staging of early cervical cancer: a prospective study and review of the literature. Clin Imaging. (2016) 40:72−8. doi: 10.1016/j.clinimag.2015.09.012 26459788

[14] Patel S Liyanage SH Sahdev A Rockall AG Reznek RH. Imaging of endometrial and cervical cancer. Insights Imaging. (2010) 1:309−28. doi: 10.1007/s13244-010-0042-7 22347925 PMC3259382

[15] Patel-Lippmann K Robbins JB Barroilhet L Anderson B Sadowski EA Boyum J. MR imaging of cervical cancer. Magn Reson Imaging Clin N Am. (2017) 25:635−49. doi: 10.1016/j.mric.2017.03.007 28668164

[16] Punwani S. Diffusion weighted imaging of female pelvic cancers: concepts and clinical applications. Eur J Radiol. (2011) 78:21−9. doi: 10.1016/j.ejrad.2010.07.028 20801592

[17] Shakur A Lee JYJ Freeman S. An update on the role of MRI in treatment stratification of patients with cervical cancer. Cancers. (2023) 15:5105. doi: 10.3390/cancers15205105 37894476 PMC10605640

[18] Shen G Zhou H Jia Z Deng H. Diagnostic performance of diffusion-weighted MRI for detection of pelvic metastatic lymph nodes in patients with cervical cancer: a systematic review and meta-analysis. Br J Radiol. (2015) 88:20150063. doi: 10.1259/bjr.20150063 PMC4651381 26111112

[19] Driscoll DO Halpenny D Johnston C Sheehy N Keogan M. 18F-FDG-PET/CT is of limited value in primary staging of early stage cervical cancer. Abdom Imaging. (2015) 40:127−33. doi: 10.1007/s00261-014-0194-x 25015401

[20] Signorelli M Guerra L Montanelli L Crivellaro C Buda A Dell’Anna T. Preoperative staging of cervical cancer: Is 18-FDG-PET/CT really effective in patients with early stage disease? Gynecol Oncol. (2011) 123:236−40. doi: 10.1016/j.ygyno.2011.07.096 21855972

[21] Chung HH Kang KW Cho JY Kim JW Park NH Song YS. Role of magnetic resonance imaging and positron emission tomography/computed tomography in preoperative lymph node detection of uterine cervical cancer. Am J Obstet Gynecol. (2010) 203:156.e1–5. doi: 10.1016/j.ajog.2010.02.041 20435285

[22] How J Boldeanu I Lau S Salvador S How E Gotlieb R. Unexpected locations of sentinel lymph nodes in endometrial cancer. Gynecol Oncol. (2017) 147:18−23. doi: 10.1016/j.ygyno.2017.07.125 28716308

[23] Bats AS Mathevet P Buenerd A Orliaguet I Mery E Zerdoud S. The sentinel node technique detects unexpected drainage pathways and allows nodal ultrastaging in early cervical cancer: insights from the multicenter prospective SENTICOL study. Ann Surg Oncol. (2013) 20:413−22. doi: 10.1245/s10434-012-2597-7 22911367

[24] Achouri A Huchon C Bats AS Bensaid C Nos C Lécuru F. Complications of lymphadenectomy for gynecologic cancer. Eur J Surg Oncol. (2013) 39:81−6. doi: 10.1016/j.ejso.2012.10.011 23117018

[25] Smits A Ten Eikelder M Dhanis J Moore W Blake D Zusterzeel P. Finding the sentinel lymph node in early cervical cancer: When is unusual not uncommon? Gynecol Oncol. (2023) 170:84−92. doi: 10.1016/j.ygyno.2022.12.013 36657244

[26] Parpinel G Laas-Faron E Balaya V Guani B Zola P Mathevet P. Survival after sentinel lymph node biopsy for early cervical cancers: a systematic review and meta-analysis. Int J Gynecol Cancer. (2023) 33:1853−60. doi: 10.1136/ijgc-2023-ESGO.130 37696646

[27] Lecuru FR McCormack M Hillemanns P Anota A Leitao M Mathevet P. SENTICOL III: an international validation study of sentinel node biopsy in early cervical cancer. A GINECO, ENGOT, GCIG and multicenter study. Int J Gynecol Cancer. (2019) 29:829−34. doi: 10.1136/ijgc-2019-000332 30898938 PMC7445752

[28] Ruscito I Gasparri ML Braicu EI Bellati F Raio L Sehouli J. Sentinel node mapping in cervical and endometrial cancer: indocyanine green versus other conventional dyes-A meta-analysis. Ann Surg Oncol. (2016) 23:3749−56. doi: 10.1245/s10434-016-5236-x 27160526

[29] Frumovitz M Plante M Lee PS Sandadi S Lilja JF Escobar PF. A randomized phase III multicenter study assessing near infrared fluorescence in the detection of sentinel lymph nodes in women with cervical and uterine cancers: the FILM Trial. Lancet Oncol. (2018) 19:1394−403. doi: 10.1016/S1470-2045(18)30448-0 30143441 PMC6580418

[30] Lührs O Ekdahl L Lönnerfors C Geppert B Persson J. Combining Indocyanine Green and Tc99-nanocolloid does not increase the detection rate of sentinel lymph nodes in early stage cervical cancer compared to Indocyanine Green alone. Gynecol Oncol. (2020) 156:335−40. doi: 10.1016/j.ygyno.2019.11.026 31780237

[31] Cabrera S Barahona-Orpinell M Almansa-González C Padilla-Iserte P Bebia V Martí L. Combined use of ICG and technetium does not improve sentinel lymph node detection in endometrial cancer: Results of the COMBITEC study. Gynecol Oncol. (2021) 162:32−7. doi: 10.1016/j.ygyno.2021.05.002 33992451

[32] Lécuru F Mathevet P Querleu D Leblanc E Morice P Daraï E. Bilateral negative sentinel nodes accurately predict absence of lymph node metastasis in early cervical cancer: results of the SENTICOL study. JCO. (2011) 29:1686−91. doi: 10.1200/JCO.2010.32.0432 21444878

[33] Cormier B Diaz JP Shih K Sampson RM Sonoda Y Park KJ. Establishing a sentinel lymph node mapping algorithm for the treatment of early cervical cancer. Gynecol Oncol. (2011) 122:275−80. doi: 10.1016/j.ygyno.2011.04.023 21570713 PMC4996075

[34] Diaz JP Gemignani ML Pandit-Taskar N Park KJ Murray MP Chi DS. Sentinel lymph node biopsy in the management of early-stage cervical carcinoma. Gynecol Oncol. (2011) 120:347−52. doi: 10.1016/j.ygyno.2010.12.334 21216450 PMC3951119

[35] Tax C Rovers MM de Graaf C Zusterzeel PLM Bekkers RLM. The sentinel node procedure in early stage cervical cancer, taking the next step; a diagnostic review. Gynecol Oncol. (2015) 139:559−67. doi: 10.1016/j.ygyno.2015.09.076 26416173

[36] Mathevet P Lécuru F Uzan C Boutitie F Magaud L Guyon F. Sentinel lymph node biopsy and morbidity outcomes in early cervical cancer: Results of a multicentre randomised trial (SENTICOL-2). Eur J Cancer. (2021) 148:307−15. doi: 10.1016/j.ejca.2021.02.009 33773275

[37] Tu H Huang H Xian B Li J Wang P Zhao W. Sentinel lymph node biopsy versus pelvic lymphadenectomy in early-stage cervical cancer: a multi-center randomized trial (PHENIX/CSEM 010). Int J Gynecol Cancer. (2020) 30:1829−33. doi: 10.1136/ijgc-2020-001857 32973117

[38] Rychlik A Angeles MA Migliorelli F Croce S Mery E Martinez A. Frozen section examination of sentinel lymph nodes can be used as a decisional tool in the surgical management of early cervical cancer. Int J Gynecol Cancer. (2020) 30:358−63. doi: 10.1136/ijgc-2019-000904 31911532

[39] Slama J Dundr P Dusek L Cibula D. High false negative rate of frozen section examination of sentinel lymph nodes in patients with cervical cancer. Gynecol Oncol. (2013) 129:384−8. doi: 10.1016/j.ygyno.2013.02.001 23395889

[40] Martínez A Mery E Filleron T Boileau L Ferron G Querleu D. Accuracy of intraoperative pathological examination of SLN in cervical cancer. Gynecol Oncol. (2013) 130:525−9. doi: 10.1016/j.ygyno.2013.01.023 23500089

[41] Papadia A Morosi C Wang J Gasparri ML Rau T Ghezzi F. SLN mapping in early-stage cervical cancer as a minimal-invasive triaging tool for multimodal treatment. Eur J Surg Oncol. (2019) 45:679−83. doi: 10.1016/j.ejso.2019.01.184 30732972

[42] Balaya V Guani B Benoit L Magaud L Bonsang-Kitzis H Ngô C. Diagnostic value of frozen section examination of sentinel lymph nodes in early-stage cervical cancer at the time of ultrastaging. Gynecol Oncol. (2020) 158:576−83. doi: 10.1016/j.ygyno.2020.05.043 32595022

[43] Roy M Bouchard-Fortier G Popa I Grégoire J Renaud MC Têtu B. Value of sentinel node mapping in cancer of the cervix. Gynecol Oncol. (2011) 122:269−74. doi: 10.1016/j.ygyno.2011.04.002 21529908

[44] Balaya V Guani B Bonsang-Kitzis H Deloménie M Ngô C Montero Macias R. Place du ganglion sentinelle dans les cancers du col utérin débutants. Bull Du Cancer. (2020) 107:696−706. doi: 10.1016/j.bulcan.2019.06.011 31627905

[45] Dundr P Cibula D Němejcová K Tichá I Bártů M Jakša R. Pathologic protocols for sentinel lymph nodes ultrastaging in cervical cancer. Arch Pathol Lab Med. (2020) 144:1011–20. doi: 10.5858/arpa.2019-0249-RA 31869245

[46] Burg LC Hengeveld EM In ‘t Hout J Bulten J Bult P Zusterzeel PLM. Ultrastaging methods of sentinel lymph nodes in endometrial cancer - a systematic review. Int J Gynecol Cancer. (2021) 31:744−53. doi: 10.1136/ijgc-2020-001964 33187974

[47] Grassi T Dell’Orto F Jaconi M Lamanna M De Ponti E Paderno M. Two ultrastaging protocols for the detection of lymph node metastases in early-stage cervical and endometrial cancers. Int J Gynecol Cancer. (2020) 30:1404–10. doi: 10.1136/ijgc-2020-001298 32376740

[48] Cibula D Kocian R Plaikner A Jarkovsky J Klat J Zapardiel I. Sentinel lymph node mapping and intraoperative assessment in a prospective, international, multicentre, observational trial of patients with cervical cancer: The SENTIX trial. Eur J Cancer. (2020) 137:69−80. doi: 10.1016/j.ejca.2020.06.034 32750501

[49] Cibula D Abu-Rustum NR Dusek L Slama J Zikán M Zaal A. Bilateral ultrastaging of sentinel lymph node in cervical cancer: Lowering the false-negative rate and improving the detection of micrometastasis. Gynecol Oncol. (2012) 127:462−6. doi: 10.1016/j.ygyno.2012.08.035 22943880

[50] Sonoda K Yahata H Okugawa K Kaneki E Ohgami T Yasunaga M. Value of intraoperative cytological and pathological sentinel lymph node diagnosis in fertility-sparing trachelectomy for early-stage cervical cancer. Oncology. (2018) 94:92−8. doi: 10.1159/000484049 29136624

[51] Dostalek L Slama J Fisherova D Kocian R Germanova A Fruhauf F. Impact of sentinel lymph node frozen section evaluation to avoid combined treatment in early-stage cervical cancer. Int J Gynecol Cancer. (2020) 30:744−8. doi: 10.1136/ijgc-2019-001113 32276933

[52] Cibula D Pötter R Planchamp F Avall-Lundqvist E Fischerova D Haie Meder C. The european society of gynaecological oncology/european society for radiotherapy and oncology/european society of pathology guidelines for the management of patients with cervical cancer. Int J Gynecol Cancer. (2018) 28:641−55. doi: 10.1097/IGC.0000000000001216 29688967

[53] Marnitz S Köhler C Affonso RJ Schneider A Chiantera V Tsounoda A. Validity of laparoscopic staging to avoid adjuvant chemoradiation following radical surgery in patients with early cervical cancer. Oncology. (2012) 83:346−53. doi: 10.1159/000341659 23006972

[54] Nasioudis D Rush M Taunk NK Ko EM Haggerty AF Cory L. Oncologic outcomes of surgical para-aortic lymph node staging in patients with advanced cervical carcinoma undergoing chemoradiation. Int J Gynecol Cancer. (2022) 32:823–7. doi: 10.1136/ijgc-2022-003394 35788115

[55] Olthof EP van der Aa MA Adam JA Stalpers LJA Wenzel HHB van der Velden J. The role of lymph nodes in cervical cancer: incidence and identification of lymph node metastases—a literature review. Int J Clin Oncol. (2021) 26:1600−10. doi: 10.1007/s10147-021-01980-2 34241726

[56] Bae BK Park SH Jeong SY Chong GO Kim MY Kim JC. Mapping patterns of para-aortic lymph node recurrence in cervical cancer: a retrospective cohort analysis. Radiat Oncol. (2021) 16:128. doi: 10.1186/s13014-021-01856-9 34246296 PMC8272280

[57] Gouy S Morice P Narducci F Uzan C Gilmore J Kolesnikov-Gauthier H. Nodal-staging surgery for locally advanced cervical cancer in the era of PET. Lancet Oncol. (2012) 13:e212–220. doi: 10.1016/S1470-2045(12)70011-6 22554549

[58] Smits RM Zusterzeel PLM Bekkers RLM. Pretreatment retroperitoneal para-aortic lymph node staging in advanced cervical cancer: a review. Int J Gynecol Cancer. (2014) 24:973−83. doi: 10.1097/IGC.0000000000000177 24978708

[59] Brockbank E Kokka F Bryant A Pomel C Reynolds K. Pre-treatment surgical para-aortic lymph node assessment in locally advanced cervical cancer. Cochrane Database Syst Rev. (2013) 2013:CD008217. doi: 10.1002/14651858.CD008217.pub3 23543561 PMC7105904

[60] Mereu L Pecorino B Ferrara M Tomaselli V Scibilia G Scollo P. Neoadjuvant chemotherapy plus radical surgery in locally advanced cervical cancer: retrospective single-center study. Cancers. (2023) 15:5207. doi: 10.3390/cancers15215207 37958381 PMC10648104

[61] Kenter GG Greggi S Vergote I Katsaros D Kobierski J van Doorn H. Randomized phase III study comparing neoadjuvant chemotherapy followed by surgery versus chemoradiation in stage IB2-IIB cervical cancer: EORTC-55994. J Clin Oncol. (2023) 41:5035−43. doi: 10.1200/JCO.22.02852 37656948

[62] McCormack M Rincón DG Eminowicz G Diez P Farrelly L Kent C. LBA8 A randomised phase III trial of induction chemotherapy followed by chemoradiation compared with chemoradiation alone in locally advanced cervical cancer: The GCIG INTERLACE trial. Ann Oncol. (2023) 34:S1276. doi: 10.1016/j.annonc.2023.10.028

[63] Leblanc E Narducci F Frumovitz M Lesoin A Castelain B Baranzelli MC. Therapeutic value of pretherapeutic extraperitoneal laparoscopic staging of locally advanced cervical carcinoma. Gynecol Oncol. (2007) 105:304−11. doi: 10.1016/j.ygyno.2006.12.012 17258799

[64] Gennigens C De Cuypere M Hermesse J Kridelka F Jerusalem G. Optimal treatment in locally advanced cervical cancer. Expert Rev Anticancer Ther. (2021) 21:657−71. doi: 10.1080/14737140.2021.1879646 33472018

[65] Tsunoda AT Marnitz S Soares Nunes J Mattos de Cunha Andrade CE Scapulatempo Neto C Blohmer JU. Incidence of histologically proven pelvic and para-aortic lymph node metastases and rate of upstaging in patients with locally advanced cervical cancer: results of a prospective randomized trial. Oncology. (2017) 92:213−20. doi: 10.1159/000453666 28142146

[66] Martinez A Voglimacci M Lusque A Ducassou A Gladieff L Dupuis N. Tumour and pelvic lymph node metabolic activity on FDG-PET/CT to stratify patients for para-aortic surgical staging in locally advanced cervical cancer. Eur J Nucl Med Mol Imaging. (2020) 47:1252−60. doi: 10.1007/s00259-019-04659-z 31915897

[67] De Cuypere M Lovinfosse P Goffin F Gennigens C Rovira R Duch J. Added value of para-aortic surgical staging compared to 18F-FDG PET/CT on the external beam radiation field for patients with locally advanced cervical cancer: An ONCO-GF study. Eur J Surg Oncol. (2020) 46:883−7. doi: 10.1016/j.ejso.2019.11.496 31784203

[68] Atri M Zhang Z Dehdashti F Lee SI Ali S Marques H. Utility of PET-CT to evaluate retroperitoneal lymph node metastasis in advanced cervical cancer: Results of ACRIN6671/GOG0233 trial. Gynecol Oncol. (2016) 142:413−9. doi: 10.1016/j.ygyno.2016.05.002 27178725 PMC4993667

[69] Selman TJ Mann C Zamora J Appleyard TL Khan K. Diagnostic accuracy of tests for lymph node status in primary cervical cancer: a systematic review and meta-analysis. CMAJ. (2008) 178:855−62. doi: 10.1503/cmaj.071124 18362381 PMC2267838

[70] Liu B Gao S Li S. A comprehensive comparison of CT, MRI, positron emission tomography or positron emission tomography/CT, and diffusion weighted imaging-MRI for detecting the lymph nodes metastases in patients with cervical cancer: A meta-analysis based on 67 studies. Gynecol Obstet Invest. (2017) 82:209−22. doi: 10.1159/000456006 28183074

[71] Choi HJ Ju W Myung SK Kim Y. Diagnostic performance of computer tomography, magnetic resonance imaging, and positron emission tomography or positron emission tomography/computer tomography for detection of metastatic lymph nodes in patients with cervical cancer: meta-analysis. Cancer Sci. (2010) 101:1471−9. doi: 10.1111/j.1349-7006.2010.01532.x 20298252 PMC11159236

[72] Gouy S Morice P Narducci F Uzan C Martinez A Rey A. Prospective multicenter study evaluating the survival of patients with locally advanced cervical cancer undergoing laparoscopic para-aortic lymphadenectomy before chemoradiotherapy in the era of positron emission tomography imaging. JCO. (2013) 31:3026−33. doi: 10.1200/JCO.2012.47.3520 23857967

[73] Leblanc E Gauthier H Querleu D Ferron G Zerdoud S Morice P. Accuracy of 18-fluoro-2-deoxy-D-glucose positron emission tomography in the pretherapeutic detection of occult para-aortic node involvement in patients with a locally advanced cervical carcinoma. Ann Surg Oncol. (2011) 18:2302−9. doi: 10.1245/s10434-011-1583-9 21347790

[74] Testa AC Di Legge A De Blasis I Moruzzi MC Bonatti M Collarino A. Imaging techniques for the evaluation of cervical cancer. Best Pract Res Clin Obstet Gynaecol. (2014) 28:741−68. doi: 10.1016/j.bpobgyn.2014.04.009 24861248

[75] Yu W Kou C Bai W Yu X Duan R Zhu B. The diagnostic performance of PET/CT scans for the detection of para-aortic metastatic lymph nodes in patients with cervical cancer: A meta-analysis. PloS One. (2019) 14:e0220080. doi: 10.1371/journal.pone.0220080 31318962 PMC6638976

[76] Moon SH Hyun SH Choi JY. Prognostic significance of volume-based PET parameters in cancer patients. Korean J Radiol. (2013) 14:1−12. doi: 10.3348/kjr.2013.14.1.1 23323025 PMC3542291

[77] Sarker A Im HJ Cheon GJ Chung HH Kang KW Chung JK. Prognostic implications of the SUVmax of primary tumors and metastatic lymph node measured by 18F-FDG PET in patients with uterine cervical cancer: A meta-analysis. Clin Nucl Med. (2016) 41:34−40. doi: 10.1097/RLU.0000000000001049 26505856

[78] Zhu Y Shen B Pei X Liu H Li G. CT, MRI, and PET imaging features in cervical cancer staging and lymph node metastasis. Am J Transl Res. (2021) 13(9):10536−44. 34650724 PMC8507065

[79] Dabi Y Simon V Carcopino X Bendifallah S Ouldamer L Lavoue V. Therapeutic value of surgical paraaortic staging in locally advanced cervical cancer: a multicenter cohort analysis from the FRANCOGYN study group. J Transl Med. (2018) 16:326. doi: 10.1186/s12967-018-1703-4 30477530 PMC6260775

[80] Marnitz S Tsunoda AT Martus P Vieira M Affonso Junior RJ Nunes J. Surgical versus clinical staging prior to primary chemoradiation in patients with cervical cancer FIGO stages IIB-IVA: oncologic results of a prospective randomized international multicenter (Uterus-11) intergroup study. Int J Gynecol Cancer. (2020) 30:1855−61. doi: 10.1136/ijgc-2020-001973 33293284 PMC7788482

[81] Frumovitz M Querleu D Gil-Moreno A Morice P Jhingran A Munsell MF. Lymphadenectomy in locally advanced cervical cancer study (LiLACS): Phase III clinical trial comparing surgical with radiologic staging in patients with stages IB2-IVA cervical cancer. J Minim Invasive Gynecol. (2014) 21:3−8. doi: 10.1016/j.jmig.2013.07.007 23911560 PMC4283493

[82] Uzan C Souadka A Gouy S Debaere T Duclos J Lumbroso J. Analysis of morbidity and clinical implications of laparoscopic para-aortic lymphadenectomy in a continuous series of 98 patients with advanced-stage cervical cancer and negative PET-CT imaging in the para-aortic area. Oncologist. (2011) 16:1021−7. doi: 10.1634/theoncologist.2011-0007 21659610 PMC3228132

[83] Petitnicolas C Azaïs H Ghesquière L Tresch-Bruneel E Cordoba A Narducci F. Morbidity of staging inframesenteric paraaortic lymphadenectomy in locally advanced cervical cancer compared with infrarenal lymphadenectomy. Int J Gynecol Cancer. (2017) 27:575−80. doi: 10.1097/IGC.0000000000000921 28166115

[84] Ouldamer L Fichet-Djavadian S Marret H Barillot I Body G. Upper margin of para-aortic lymphadenectomy in cervical cancer. Acta Obstet Gynecol Scand. (2012) 91:893−900. doi: 10.1111/j.1600-0412.2012.01443.x 22553934

[85] Leblanc E Katdare N Narducci F Bresson L Gouy S Morice P. Should systematic infrarenal para-aortic dissection be the rule in the pretherapeutic staging of primary or recurrent locally advanced cervix cancer patients with a negative preoperative para-aortic PET imaging? Int J Gynecol Cancer. (2016) 26:169−75. doi: 10.1097/IGC.0000000000000588 26569062

[86] Vergote I Amant F Berteloot P Van Gramberen M. Laparoscopic lower para-aortic staging lymphadenectomy in stage IB2, II, and III cervical cancer. Int J Gynecol Cancer. (2002) 12:22−6. doi: 10.1136/ijgc-00009577-200201000-00004 11860532

[87] Gil-Moreno A Magrina JF Pérez-Benavente A Díaz-Feijoo B Sánchez-Iglesias JL García A. Location of aortic node metastases in locally advanced cervical cancer. Gynecol Oncol. (2012) 125:312−4. doi: 10.1016/j.ygyno.2012.02.008 22333995

[88] Koh WJ Abu-Rustum NR Bean S Bradley K Campos SM Cho KR. Cervical cancer, version 3.2019, NCCN clinical practice guidelines in oncology. J Natl Compr Canc Netw. (2019) 17:64−84. doi: 10.6004/jnccn.2019.0001 30659131

[89] Querleu D Dargent D Ansquer Y Leblanc E Narducci F. Extraperitoneal endosurgical aortic and common iliac dissection in the staging of bulky or advanced cervical carcinomas. Cancer. (2000) 88:1883−91. doi: 10.1002/(SICI)1097-0142(20000415)88:8<1883::AID-CNCR18>3.0.CO;2-3 10760766

[90] Dargent D Ansquer Y Mathevet P. Technical development and results of left extraperitoneal laparoscopic paraaortic lymphadenectomy for cervical cancer. Gynecol Oncol. (2000) 77:87−92. doi: 10.1006/gyno.1999.5585 10739695

[91] Kusunoki S Huang KG Magno A Lee CL. Laparoscopic technique of para-aortic lymph node dissection: A comparison of the different approaches to trans- versus extraperitoneal para-aortic lymphadenectomy. Gynecol Minim Invasive Ther. (2017) 6:51−7. doi: 10.1016/j.gmit.2016.01.003 30254875 PMC6113969

[92] Nemejcova K Kocian R Kohler C Jarkovsky J Klat J Berjon A. Central pathology review in SENTIX, a prospective observational international study on sentinel lymph node biopsy in patients with early-stage cervical cancer (ENGOT-CX2). Cancers (Basel). (2020) 12:1115. doi: 10.3390/cancers12051115 32365651 PMC7281480

[93] Ronsini C Solazzo MC Molitierno R De Franciscis P Pasanisi F Cobellis L. Fertility-sparing treatment for early-stage cervical cancer ≥ 2 cm: can one still effectively become a mother? A systematic review of fertility outcomes. Ann Surg Oncol. (2023) 30:5587−96. doi: 10.1245/s10434-023-13542-z 37261562 PMC10409841

[94] Amant F Berveiller P Boere IA Cardonick E Fruscio R Fumagalli M. Gynecologic cancers in pregnancy: guidelines based on a third international consensus meeting. Ann Oncol. (2019) 30:1601−12. doi: 10.1093/annonc/mdz228 31435648

[95] Ishiguro T Nishikawa N Ishii S Yoshihara K Haino K Yamaguchi M. PET/MR imaging for the evaluation of cervical cancer during pregnancy. BMC Pregnancy Childbirth. (2021) 21:288. doi: 10.1186/s12884-021-03766-w 33838651 PMC8037915

[96] Ma L Yu S Xu X Moses Amadi S Zhang J Wang Z. Application of artificial intelligence in 3D printing physical organ models. Mater Today Bio. (2023) 23:100792. doi: 10.1016/j.mtbio.2023.100792 PMC10511479 37746667

[97] Jiang Y Wang C Zhou S. Artificial intelligence-based risk stratification, accurate diagnosis and treatment prediction in gynecologic oncology. Semin Cancer Biol. (2023) 96:82−99. doi: 10.1016/j.semcancer.2023.09.005 37783319

[98] Lambin P Rios-Velazquez E Leijenaar R Carvalho S van Stiphout RGPM Granton P. Radiomics: Extracting more information from medical images using advanced feature analysis. Eur J Cancer. (2012) 48:441−6. doi: 10.1016/j.ejca.2011.11.036 22257792 PMC4533986

[99] Li L Zhang J Zhe X Tang M Zhang X Lei X. A meta-analysis of MRI-based radiomic features for predicting lymph node metastasis in patients with cervical cancer. Eur J Radiol. (2022) 151:110243. doi: 10.1016/j.ejrad.2022.110243 35366583

[100] Lucia F Bourbonne V Pleyers C Dupré PF Miranda O Visvikis D. Multicentric development and evaluation of 18F-FDG PET/CT and MRI radiomics models to predict para-aortic lymph node involvement in locally advanced cervical cancer. Eur J Nucl Med Mol Imaging. (2023) 50:2514−28. doi: 10.1007/s00259-023-06180-w 36892667

